# Coelonin, an Anti-Inflammation Active Component of *Bletilla striata* and Its Potential Mechanism

**DOI:** 10.3390/ijms20184422

**Published:** 2019-09-08

**Authors:** Fusheng Jiang, Meiya Li, Hongye Wang, Bin Ding, Chunchun Zhang, Zhishan Ding, Xiaobo Yu, Guiyuan Lv

**Affiliations:** 1 College of Life Science, Zhejiang Chinese Medical University, Hangzhou 310053, China; jfs1020@163.com (F.J.); db@zcmu.edu.cn (B.D.); 2 Academy of Chinese Medical Sciences, Zhejiang Chinese Medical University, Hangzhou 310053, China; lmeiya@126.com; 3 State Key Laboratory of Proteomics, Beijing Proteome Research Center, National Center for Protein Sciences (PHOENIX Center, Beijing), Beijing Institute of Lifeomics, Beijing 102206, China; Crystalhongye@126.com; 4 College of Pharmaceutical Science, Zhejiang Chinese Medical University, Hangzhou 310053, China; zhangchun200123@163.com; 5 College of Medical Technology, Zhejiang Chinese Medical University, Hangzhou 310053, China; zjtcmdzs@163.com

**Keywords:** coelonin, *Bletilla striata*, anti-inflammation, signal pathway, cell-cycle arrest, PTEN

## Abstract

Ethanol extract of *Bletilla striata* has remarkable anti-inflammatory and anti-pulmonary fibrosis activities in the rat silicosis model. However, its active substances and molecular mechanism are still unclear. To uncover the active ingredients and potential molecular mechanism of the *Bletilla striata* extract, the lipopolysaccharide (LPS)-induced macrophage inflammation model and phospho antibody array were used. Coelonin, a dihydrophenanthrene compound was isolated and identified. It significantly inhibited LPS-induced interleukin-1β (IL-1β), interleukin-6 (IL-6) and tumor necrosis factor-α (TNF-α) expression at 2.5 μg/mL. The microarray data indicate that the phosphorylation levels of 32 proteins in the coelonin pre-treated group were significantly down-regulated. In particular, the phosphorylation levels of the key inflammatory regulators factor nuclear factor-kappa B (NF-κB) were significantly reduced, and the negative regulator phosphatase and tensin homologue on chromosome ten (PTEN) was reduced. Moreover, the phosphorylation level of cyclin dependent kinase inhibitor 1B (p27^Kip1^), another downstream molecule regulated by PTEN was also reduced significantly. Western blot and confocal microscopy results confirmed that coelonin inhibited LPS-induced PTEN phosphorylation in a dose-dependent manner, then inhibited NF-κB activation and p27^Kip1^ degradation by regulating the phosphatidylinositol-3-kinases/ v-akt murine thymoma viral oncogene homolog (PI3K/AKT) pathway negatively. However, PTEN inhibitor co-treatment analysis indicated that the inhibition of IL-1β, IL-6 and TNF-α expression by coelonin was independent of PTEN, whereas the inhibition of p27^Kip1^ degradation resulted in cell-cycle arrest in the G1 phase, which was dependent on PTEN. The anti-inflammatory activity of coelonin in vivo, which is one of the main active ingredients of *Bletilla striata*, deserves further study.

## 1. Introduction

*Bletilla striata* (Thunb.) Reichb.f is a famous traditional Chinese herb that is widely used in the treatment of lung and stomach diseases such as pneumogastric hemorrhage, silicosis, tuberculosis, and gastric ulcer; it can also be used for the treatment of skin cracks, burns and freckles when combined with other traditional Chinese medicines. Numerous compounds have been identified from *Bletilla striata*, such as benzyls, phenanthrenes, dihydrophenanthrenes, anthracene, phenolic acid and polysaccharides [1,2]. Among these, polysaccharides are the most extensively and deeply studied, and their anti-ulcer [3], wound healing [4], homeostasis [5] and immune regulation [6] effects have represented most of the efficacy of *Bletilla striata*. However, in recent years, the pharmacological activities of the small molecular components in *Bletilla striata* have also attracted much attention. Liu [7] reported that the 80% ethanol elunt fraction of D101 macroporous resin significantly reduced bleeding time and increased the maximum platelet aggregation rate. Our previous research showed that the ethanol extract of *Bletilla striata* dose dependently inhibited alcohol induced gastric ulcer and silica induced silicosis in rats [8,9]. Furthermore, the ethanol extract of *Bletilla striata* significantly down regulated the serum level of IL-1β, TNF-α, transforming growth factor-β (TGF-β) and other inflammatory factors in rats with silicosis [9], thereby reducing the degree of pulmonary fibrosis, and this effect is far more effective than the polysaccharide of *Bletilla striata* [10]. However, its active components and underlying molecular mechanisms are unclear.

Silicosis is a type of systemic disease, characterized by chronic persistent inflammation and progressive fibrosis in lung tissue. The innate immune response mediated by alveolar macrophage plays a very important role in inflammatory reaction during the process of silicosis. The activated macrophages release proinflammatory mediators such as IL-6, IL-1β, TNF-α, TGF-β and platelet-derived growth factor (PDGF), etc. [11]. These inflammatory factors are recognized as key factors in pulmonary fibrosis, and the interruption of these factor pathways can alleviate or prevent fibrosis [12,13,14]. The classic LPS-induced RAW264.7 macrophage model can mimic the process of macrophage activation in vitro. One active compound 2,7-dihydroxy-4-methoxy-9,10-dihydrophenanthrene (coelonin) from *Bletilla striata* was separated and identified under the guidance of this cell model and combined with column chromatography.

Although few studies have described the anti-inflammatory effect of coelonin, but we found that this compound significantly down regulated IL-1β and IL-6 expression at 2.5 μg/mL on LPS-induced RAW264.7 cell. Hence, coelonin may be one of the main active components contributing to the anti-silicosis effect of *Bletilla striata*. In this study, we used a Phospho Explorer Antibody Array PEX100 to discover the potential target of the anti-inflammation effect of coelonin. The microarray results imply that coelonin may play an anti-inflammatory and cell-cycle regulation role through the PTEN/AKT pathway. Examining the down-stream signaling profile and cytokines secretion in RAW264.7 cells induced by LPS with or without coelonin or the PTEN inhibitor SF1670 suggests that coelonin blocked RAW264.7 cells in the G1 phase cell cycle in a PTEN- dependent manner, and PTEN may partially participated in coelonin inhibition on the secretion of inflammatory factors. Therefore, the potential molecular mechanism of the anti-inflammatory effect of coelonin remains to be addressed. Furthermore, as one of the main active ingredients of *Bletilla striata*, the anti-inflammatory activity of coelonin in vivo deserves further study.

## 2. Results

### 2.1. Separation, Purification and Identification of Active Components from Bletilla striata

**Figure 1:**
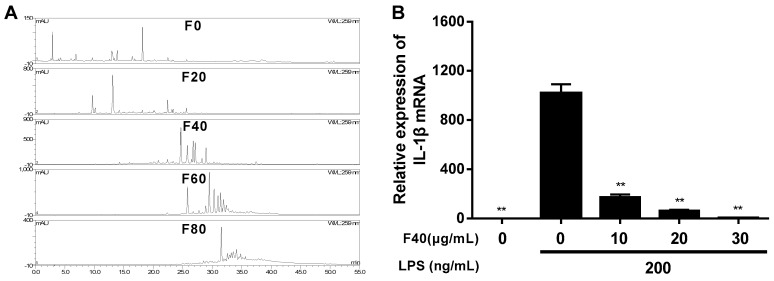
(**A**) HPLC characterization of the five fractions. A total of 10 μL each sample (1 mg/mL) was injected and analyzed using a Dionex UltiMate^TM^ 3000 HPLC system with photodiode array detection (PAD) at 259 nm. A Symmetrix ODS-RC18 (25 × 4.6 mm, 5 mm) HPLC column protected with a Phenomenex security guard column (C18, 4 × 3.0 mm) operated at 30 °C was used, and the flow rate was maintained at 1 mL/min. The elution solvents were acetonitrile (a) and 0.1% acetic acid (b). Samples were eluted according to the following gradient: 0–35 min 30% a isocratic, 35–45 min 30% to 40% a, 45–55 min 40% a isocratic, and finally washing and recondition of the column. (**B**) Relative expression of IL-1β mRNA after treatment with F40. RAW264.7 cells were pretreated with different concentration of F40 for 1 h and then treated with 200 ng/mL LPS for 6 h. Total RNA was extracted and genes expression level were analyzed by RT-PCR in triplicate. The expression level of each gene was normalized to glyceraldehyde-3-phosphate dehydrogenase (GAPDH) mRNA. Data are expressed as mean ± SD (*n* = 6). ** *p* < 0.01 vs. LPS treatment group.

The ethanol extraction of *Bletilla striata* tuber was separated into five fractions using the polyamide adsorption method, then they were characterized by the high performance liquid chromatography (HPLC) method (see Figure 1A). The results indicated that there were few common peaks in each fraction, which shows the effective enrichment effect of the polyamide column. The anti-inflammation activity of the five fractions was screened on the LPS-induced RAW264.7 cell model, and the real-time polymerase chain reaction (RT-PCR) results indicate that except F0 and F80, the fractions dose-dependently inhibited IL-1β expression, whereas F80 showed inhibition activity at low dosage, but the messenger RNA (mRNA) expression level of IL-1β was dose-dependently increased to even higher than the LPS-treated group at 30 μg/mL (see Supplementary Figure S1). F40 showed remarkable inhibition activity and 83.07% of IL-1β mRNA expression was inhibited at a concentration of 10 μg/mL (see Figure 1B).

**Figure 2:**
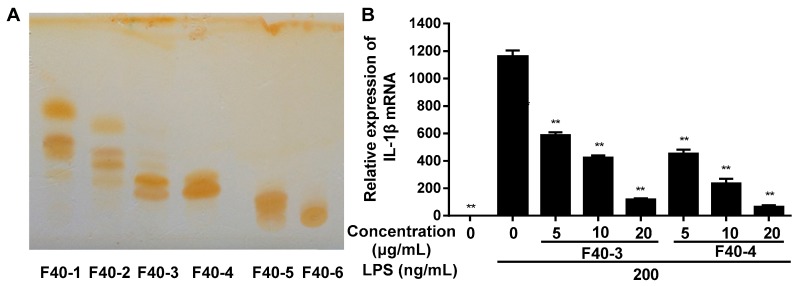
(**A**) Sub-fractions separated from F40 by silica gel chromatography. TLC was performed on precoated silica gel 60 F254 plates (Qingdao Haiyang Chemical Co., Ltd., Qingdao, China), developed with chloroform-methanol (4:0.1, V/V) and then exposed to the iodine vapor in a dark enclosed chamber for 10 min. (**B**) The relative expression of IL-1β mRNA after treatment with sub-fractions of F40-3 and F40-4. RAW264.7 cells were pretreated with different concentration of different sub-fractions of F40 for 1 h, then treated with 200 ng/mL LPS for 6 h. Total RNA was extracted and genes expression levels were analyzed by RT-PCR in triplicate. The expression level of each gene was normalized to GAPDH mRNA. Data are expressed as mean ±SD (*n* = 6). ** *p* < 0.01 vs. LPS treatment group.

**Figure 3:**
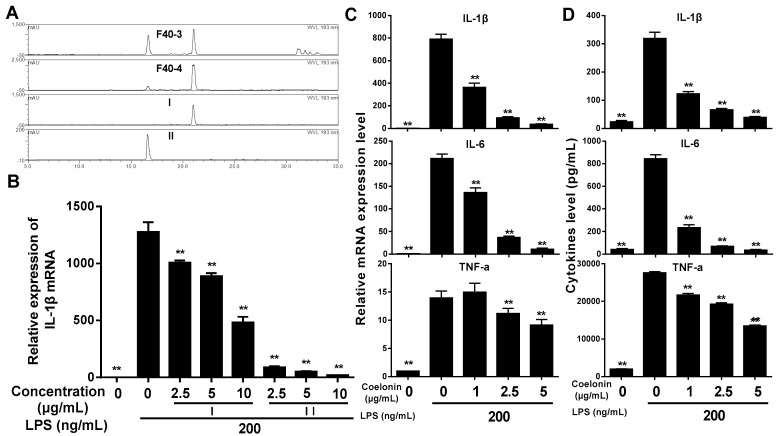
(**A**) HPLC characterization of the sub-fractions F40-3, F40-4 and two purified compounds. 10 μL each sample (0.1 mg/mL) was injected and analyzed using a Dionex UltiMate^TM^ 3000 HPLC system with PAD at 193 nm. A Symmetrix ODS-RC18 (25 × 4.6 mm, 5 mm) HPLC column protected with a Phenomenex security guard column (C18, 4 × 3.0 mm) operated at 30 °C was used, and the flow rate was maintained at 1 mL/min. The elution solvents were acetonitrile (a) and 0.1% acetic acid (b). Samples were eluted according to the following gradient: 0–5 min 10% to 35% a, 5–12 min 35% a isocratic, 12–16 min 35% to 45% a, 16–22 min 45% a isocratic, 22–35 min 45% to 80% a, and finally washing and recondition of the column. (**B**) The relative expression of IL-1β mRNA treated by active compounds. (**C**) Inhibitory effect of coelonin on LPS-induced gene expression in RAW264.7 macrophages. RAW264.7 cells were pretreated with different concentration of compound I or II for 1 h, then stimulated with 200 ng/mL of LPS for 6 h. Total RNA was extracted and genes expression level were analyzed by RT-PCR in triplicate. (**D**) Inhibitory effect of coelonin on LPS-induced cytokine secretion in RAW264.7 macrophages. RAW264.7 cells were pretreated with coelonin for 1 h and then treated with 200 ng/mL LPS. 12 h after LPS stimulation, culture supernatants was collected for IL-6 and TNF-α detection by cytometric bead array (CBA) method; for IL-1β detection, the remaining cells were following treated by 1 mM adenosine triphosphate (ATP) for an additional 15 min at 37 °C [18], then supernatants were collected and analyzed by the CBA method in triplicate. Data are expressed as mean ± SD (*n* = 6). ** *p* < 0.01 vs. LPS treatment group.

The F40 fraction was further separated by silica gel chromatography. Dry silica gel was packed into a glass column (diameter via height ratio 1:10), then dry sample (sample silica gel ratio 1:3) was loaded and eluted with chloroform-methanol (50:1, V/V) at 2 mL/min. Fractions of 10 mL were collected and monitored by thin-layer chromatography (TLC), visualized by iodine vapor and those possessing similar Rf values were combined and six sub-fractions of F40 were obtained (see Figure 2A). Anti-inflammation assay manifested that all sub-fractions inhibited the expression of IL-1β in a dose-dependent manner (see Supplementary Figure S2), and the sub-fractions of F40-3 and F40-4 showed significant inhibition ratio at 20 μg/mL (see Figure 2B). HPLC analysis revealed that F40-3 and F40-4 were mainly composed of two identical compounds (see Figure 3A), and the two compounds were purified by Dionex UltiMate^TM^ 3000 semi-prepared HPLC system. A Welch Ultimate^®^ XB-C18 (10 × 250 mm, 10 μm) HPLC column operated at 30 °C was used, and the flow rate was maintained at 5 mL/min. Samples were isocratic eluted with acetonitrile (30%) and 0.1% acetic acid (70%).

The results of mass spectrometry (MS) and nuclear magnetic resonance (NMR) spectra are as follows: Compound I (HPLC > 98%) electrosprary ionization-mass spectrometry (ESI-MS) *m/z*: 245.1108 (M + H)^+^. ^1^H-NMR (CD_3_OD) δ: 2.79 (4H, m, CH_2_), 3.71 (3H, s, OCH_3_), 6.19 (1H, dd, *J* = 1.8, 2.4 Hz, H-4), 6.25 (2H, dd, *J* = 1.8, 1.8 Hz, H-2, 6), 6.63 (3H, m, H-2’, 4’, 6’), 7.08 (1H, dd, *J* = 7.8, 8.4 Hz, H-5’); ^13^C-NMR (CD_3_OD) δ: 160.83 (C-5), 157.98 (C-3), 156.93 (C-3’), 144.06 (C-1), 143.29 (C-1’), 128.84 (C-5’), 119.43 (C-6’), 114.93 (C-2’), 112.36 (C-4’), 107.63 (C-2), 105.12 (C-6), 98.54 (C-4), 54.10 (OCH_3_), 37.80 (CH2-a’), 37.47 (CH_2_-a). Compared with the data shown in literature [15], the compound was identified as batatasin III. Compound II (HPLC > 98%) ESI-MS *m/z*: 243.1006 (M + H)^+^. ^1^H-NMR (CD_3_OD) δ: 2.64 (4H, s, H-9, 10), 3.83 (3H, s, 4-OCH_3_), 6.32 (1H, d, *J* = 2.4 Hz, H-1), 6.41 (1H, d, *J* = 2.4 Hz, H-3), 6.64 (1H, d, *J* = 1.8 Hz, H-6), 6.63 (1H, dd, *J* = 6, 2.4 Hz, H-8), 8.02 (H, d, *J* = 9 Hz, H-5). ^13^C-NMR (CD_3_OD) δ: 157.68 (C-2), 154.63 (C-7), 156.04 (C-4), 140.42 (C-10a), 139.08 (C-8a), 128.63 (C-5), 113.61 (C-4a), 124.77 (C-5a), 112.17(C-6), 115.37 (C-8), 106.90 (C-1), 97.83 (C-3), 54.45 (-OCH_3_), 29.80 (C-9), 30.38 (C-10). Comparison with the data shown in literature [16], led to the compound being identified as coelonin.

The anti-inflammation activity was verified (see Figure 3B) and both coelonin and batatasin III showed dose-dependent inhibition activity. A total of 93.1% of IL-1β mRNA expression was inhibited by coelonin at 2.5 μg/mL, which was significantly better than that of batatasin III at 10 μg/mL (62.3%). This result implies that coelonin is probably the main anti-inflammatory component of *Bletilla striata*.

### 2.2. Inhibitory Effect of Coelonin on LPS-Induced IL-1β, IL-6, TNF-α Gene Expression and Protein Secretion in RAW264.7 Macrophages

LPS can induce the expression and secretion of a variety of inflammatory factors, such as IL-1β, IL-6, TNF-*α*, monocyte chemo-attractant protein-1 (MCP-1), inducible nitric oxide synthase (iNOS) and cyclooxygenase 2 (COX2) et al. Using IL-1β as an index, a highly active ingredient, coelonin, was isolated from *Bletilla striata* by the RAW264.7 cell inflammation model. Here, two other important inflammatory factors, IL-6 and TNF-α were examined as well. We confirmed that coelonin was without a significant cytotoxic at a concentration of up to 5 μg/mL [17] (see Supplementary Figures S3 and S4). LPS markedly elevated IL-1β, IL-6 and TNF-*α* mRNA expression and protein secretion, but coelonin dose-dependently lowered these levels in macrophages (see Figure 3C,D). These results confirmed the anti-inflammation effect of coelonin. However, there was almost no anti-inflammation report of this compound; therefore, the underlying mechanism is worth further research.

### 2.3. Identifying Differentially Expressed Protein Phosphorylation Sites Induced by Coelonin Treatment

**Figure 4:**
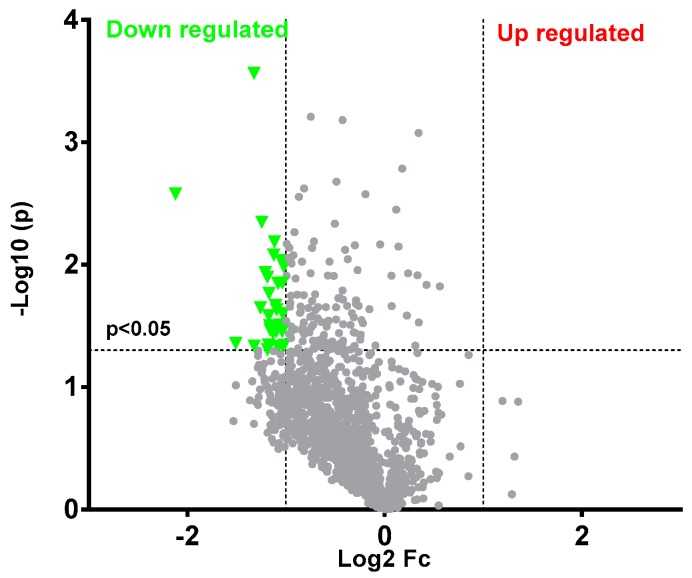
Protein and phosphorylation altered after coelonin treatment. From a total of 1318 differentially phosphorylated proteins, 32 were selected as significantly downregulated by coelonin using a volcano plot analysis.

To identify differentially expressed signaling-associated phosphorylated proteins between coelonin-treated and untreated RAW264.7 cells induced by LPS, the expression levels of phospho-antibody specific proteins were compared. Of the 1318 antibodies analyzed in microarray experiments, a total of 32 different phosphorylation proteins showed downregulated expression using a fold ratio ≥2 as the cutoff criterion (Figure 4, Table 1). In addition, we performed a protein network analysis using REACTOME (http://reactome.ncpsb.org/) to identify major interactions, three major cellular processes were significantly enriched (*p* value < 0.05) as follows: (1) Immune system, (2) signal transduction, and (3) cell cycle. LPS is the component of the outer membrane of Gram-negative bacteria, and is one of the most well characterized pathogen-associated molecular patterns (PAMPs), which can be recognized by Toll-like receptor 4 (TLR4), and trigger innate responses [19], such as enhancing the secretion of cytokines and chemokines, and promoting macrophages migration and proliferation [20]. Therefore, it is evident that the protein network analysis results imply coelonin may block LPS induced RAW264.7 cell signal transduction, immune response, and proliferation.

**Figure 5:**
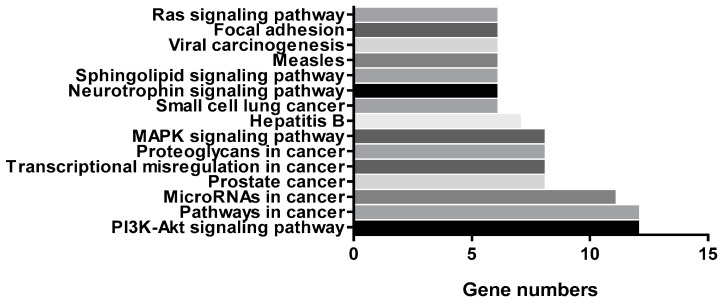
Number of genes in signaling pathways changed by coelonin pretreatment.

Moreover, we entered the ENTREZ Gene IDs of the 32 genes into the DAVID Bioinformatics Resources 6.8 database. As depicted in Figure 5, the numbers of changed genes in the PI3K/AKT signaling pathway, the mitogen-activated protein kinase (MAPK) signaling pathway, the neurotrophin signaling pathway, the Sphingolipid signaling pathway and Ras signaling pathway were ranked in the top 15. In addition, we found that within these genes, the maximum number of genes belonged to the PI3K/AKT signal pathway. It is noteworthy that the phosphorylation of both subunits p65 and p105/50 of transcription factor NF-κB in this signaling pathway was reduced, which was closely related to the regulation of inflammatory gene expression [21].

### 2.4. Validation of Proteomic Findings–Coelonin Treatment Inhibits Inflammatory Cytokines Secretion by Blocking NF-κB Activation

**Figure 6:**
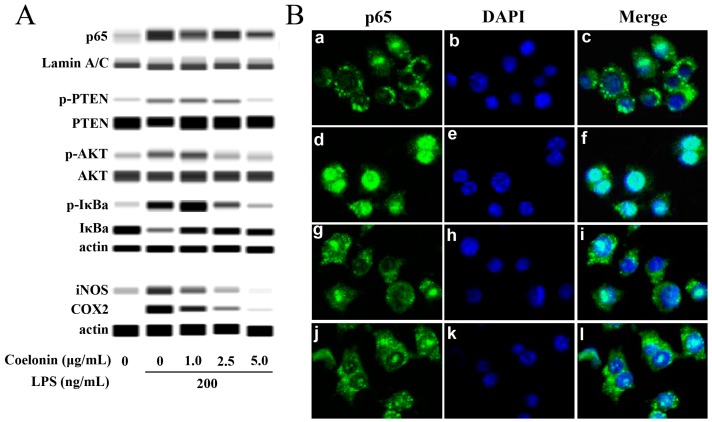
(**A**) Coelonin inhibits LPS induced NF-κB activation in macrophage. RAW264.7 cells were pretreated with coelonin for 1 h and then treated with 200 ng/mL LPS for 30 min. Then, the nuclear protein was extracted, the content of p65 in the nucleus was determined, the total cellular protein was extracted, and the levels of phosphorylated PTEN, AKT and IκBα were determined. For iNOS and COX2 detection, RAW264.7 cells were pretreated with coelonin for 1 h, then treated with 200 ng/mL LPS for 24 h, then total cellular protein was extracted and analyzed. (**B**) Confocal microscopy analysis of p65 nucleus translocation. RAW264.7 cells were incubated with solvent (a–c) or 200 ng/mL LPS for 1 h in the absence (d–f) or presence 2 μM of the NF-κB inhibitor APDC (g–i) or 5 μg/mL of coelonin (j–l).

NF-κB is the most important signaling molecule induced by LPS through TLR4. The phosphorylated NF-κB can translocate to the nucleus, interacts with the κB elements and cause numerous cytokines secretion such as IL-1β, MCP-1 and TNF-α et al. [21]. As observed in Table 1, both NF-κB p65 and p105/50 phosphorylation levels were significantly reduced by 2.5 μg/mL coelonin treatment. Therefore, we performed western blot and immunofluorescence assays by nucleus translocation of p65 for validation. As shown in Figure 6A, the western blot results indicated that LPS stimulation significantly increased p65 accumulation in the nucleus, but the coelonin dose dependently reduced the effect of LPS. Moreover, confocal microscopic analysis reconfirmed that LPS stimulation significantly induced p65 translocation from the cytoplasm to the nucleus, which was remarkably inhibited by 2 μM of ammonium pyrrolidine dithiocarbamate (APDC), an inhibitor of NF-κB, and 5.0 μg/mL of coelonin pre-treatment (Figure 6B). In addition to cytokines, numerous studies have shown that NF-κB also regulates the expression of iNOS and COX2 genes [22,23]. Furthermore, over expression of iNOS and COX2 can lead to inflammation, tissue damage and even tumorigenesis [24,25]. As observed in Figure 6A, 200 ng/mL LPS treatment for 24 h caused a remarkable increased expression of iNOS and COX2. Pre-treatment with coelonin dose dependently reversed LPS-induced iNOS and COX2 expression. These results were consistent with the microarray results and confirmed that coelonin exerts its anti-inflammatory effect by inhibiting NF-κB activity.

### 2.5. Colonin May Partially Inhibit the Activation of NF-κB through PTEN/AKT Pathway

**Figure 7:**
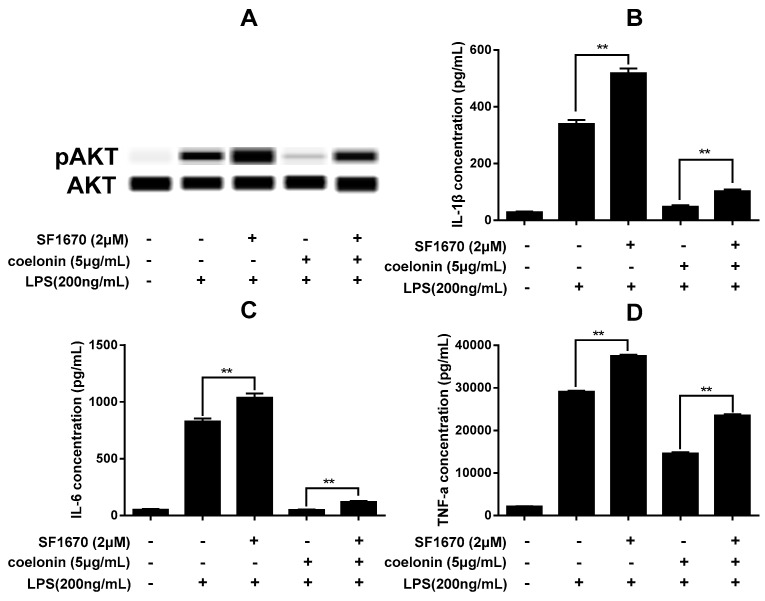
(**A**) Western blotting result of pAKT. RAW264.7 cells were incubated with solvent or 200 ng/mL LPS for 30 min in the absence or presence 5 μg/mL of coelonin or 2 μM of the PTEN inhibitor SF1670 or 2 μM of SF1670 combined with 5 μg/mL of coelonin. Then total cellular protein was extracted and analyzed by a simple western immunoblotting technique on a Peggy Sue system. (**B**–**D**) Effect of PTEN inhibitor SF1670 on anti-inflammatory activity of coelonin. RAW264.7 cells were incubated with solvent or 200 ng/mL LPS for 12 h in the absence or presence 2 μM of the PTEN inhibitor SF1670 or 5 μg/mL of coelonin or 2 μM of SF1670 combined with 5 μg/mL of coelonin. Culture supernatants was collected for IL-1β, IL-6 and TNF-α detection by CBA method. Data are expressed as mean ± SD (*n* = 6). ** *p* < 0.01.

It is well know that LPS can activate NF-κB through the Toll-like receptor 4/myeloid differentiation factor 88/IL-1 receptor associated kinase/TNF receptor associated factor 6/TGF beta-Activated Kinase 1/inhibitor of nuclear factor-κB kinase (TLR4/MyD88/IRAK/TRAF6/TAK1/IKKs) pathway [26] and now, several lines of evidence suggest that LPS can also activate NF-κB through the TLR4/MyD88/PI3K/AKT/IKKs pathway [27,28]. However, the PI3K/AKT pathway is negatively regulated by the PTEN [29,30]. Previous research has shown that the down regulation of PTEN can activate NF-κB activity by increasing p65 nucleus translocation in mouse mesangial cells and bovine alveolar macrophages, contrary, activate PTEN would reverse the effect [29,31]. The microarray results indicated that coelonin treatment significantly down-regulated the phosphorylation of PTEN at Ser380/Thr382/Thr383 (see Table 1), which implies that coelonin may inactivate NF-κB by restoring the activity of PTEN, as phosphorylation of PTEN will make it inactivated [30]. Thus, western blotting was carried out to verify this presumption. As show in Figure 6A, LPS significantly increased the phosphorylation of PTEN, AKT and inhibitor of NF-κB (IκBa), which was dose-dependently reduced by coelonin pre-treatment (Figure 6A). In order to further confirm the possible inhibition activity of coelonin against LPS-induced NF-κB activation mediated by PTEN/AKT pathway, RAW264.7 cells were pre-treated with PTEN inhibitor SF1670. As show in Figure 7A, pre-treatment with SF1670 significantly increased LPS-induced AKT phosphorylation, which could not be downregulated by additional coelonin. Meanwhile, SF1670 also dramatically promoted LPS-induced secretion of IL-1β, IL-6 and TNF-α, and significantly reduced but could not completely block the inhibitory activity of coelonin. This result further indicates that PTEN did participate in PI3K/AKT/NF-κB activation pathway. However, it is note worthy that most levels of IL-1β and IL-6 were still significantly inhibited by coelonin co-treated with SF1670, suggesting that more critical pathways need to be identified besides the PTEN/AKT pathway.

### 2.6. Coelonin Treatment Leads to G1 Cell Cycle Arrest through PTEN

**Figure 8:**
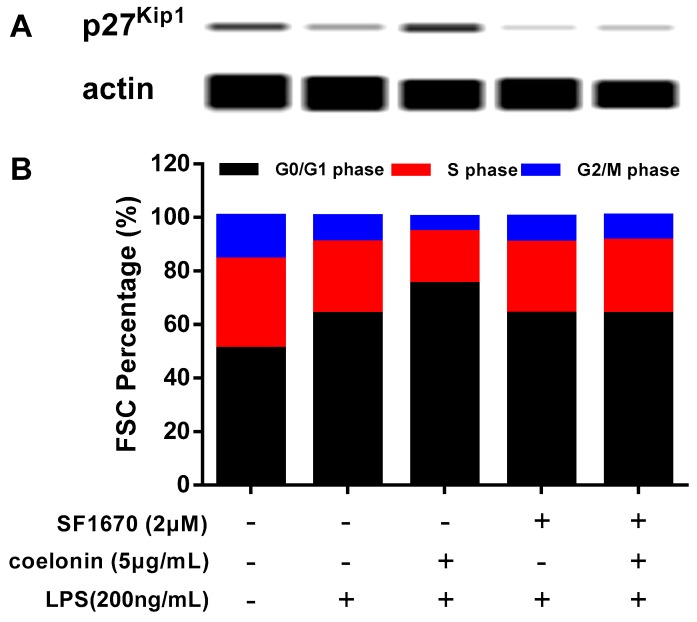
(**A**) Western blotting result of p27^kip1^. RAW264.7 cells were incubated with solvent or 200 ng/mL LPS for 12 h in the absence or presence 5 μg/mL of coelonin or 2 μM of the PTEN inhibitor SF1670 or 2 μM of SF1670 combined with 5 μg/mL of coelonin. Then total cellular protein was extracted and analysed by a simple western immunoblotting technique on a Peggy Sue system. (**B**) Coelonin induce G1 cell cycle arrest through PTEN analysed by flow cytometry. RAW264.7 cells were incubated with solvent or 200 ng/mL LPS for 12 h in the absence or presence 5 μg/mL of coelonin or 2 μM of the PTEN inhibitor SF1670 or 2 μM of SF1670 combined with 5 μg/mL of coelonin. Cells were harvested, fixed and stained with PI, and cell cycle distributions were analyzed on a BD Accuri™ C6 flow cytometer in triplicate.

The PI3K-Akt pathway has been shown be involved in a variety of cellular processes, including inflammation response [27], cell survival and proliferation [32]. Many reports indicate that the PI3K/AKT pathway plays a pivotal role in regulating cell cycle progression through phosphorylation and degradation of cell cycle regulator p27^Kip1^ [33,34]. P27^Kip1^ can interact with cyclin-dependent kinase 2 (CDK2) and cyclinE to prevent cell entry into the S phase, and over-expression p27^Kip1^ would promote cell G1 phase cell cycle arrest [35]. In many cancer cells, such as cervical cancer cells, oral squamous cell carcinoma cells, and prostatic carcinoma cells, the expression protein of p27^Kip1^ was significantly reduced, the invasion and migration ability was enhanced, and the expression of p27^Kip1^ was negatively correlated with survival [33,36]. We noticed that the microarray results showed that coelonin significantly down regulated the phosphorylation of p27^kip1^, and interestingly, a previous study indicated that the up-regulation of PTEN prevents p27^Kip1^ phosphorylation and proteolysis [37]. Hence, we speculate that coelonin may inhibit p27^Kip1^ phosphorylation and degradation mediated by PTEN. Western blot confirmed that 5 μg/mL coelonin treatment significantly recovered the p27^Kip1^ level, which was even higher than the un-treatment group, while LPS stimulation significantly reduced the p27^Kip1^ level, and PTEN inhibitor SF1670 almost completely abrogated the effect of coelonin (see Figure 8A). Theoretically, high levels of p27^Kip1^ can block cells in the G1 phase cell-cycle, so we used flow cytometry to verify whether coelonin could induce G1 phase cell cycle arrest in RAW264.7 cells. As observed in Figure 8B, coelonin pre-treatment remarkably induced G1 phase cell cycle arrest and SF1670 completely inhibited the effect of coelonin. These data suggest that coelonin inhibits p27^Kip1^ degradation in a PTEN-dependent manner, thereby exerting its G1 phase cell cycle arrest effect in RAW264.7 cells.

## 3. Discussion

To date, over 200 phenanthrene compounds have been isolated and identified, most of which come from the Orchidaceae family [38]. In *Bletilla striata*, a member of Orchidaceae, more than 30 phenanthrenes have been isolated from its pseudobulbs and fibrous roots, most of which showed antitumor and antimicrobial activities [39,40]. In addition, numerous studies have shown that phenanthrene and dihydrophenanthrene derivatives with remarkable anti-inflammation activity [38,41,42]. In this study, coelonin, a compound with a strong anti-inflammatory activity, was isolated and identified from the pseudobulbs of *Bletilla striat* under the guidance of biological activity screening. Few studies have reported its anti-inflammatory activity, and our studies show that coelonin can dose-dependently inhibit LPS-induced expression and secretion of IL-1β, IL-6 and TNF-α in RAW264.7 cells. Furthermore, we found that *Bletilla striata* has a high content of coelonin (0.020%–0.301% in different samples) [43]. Therefore, coelonin is probably one of the main anti-inflammatory active components of *Bletilla striata*.

**Figure 9:**
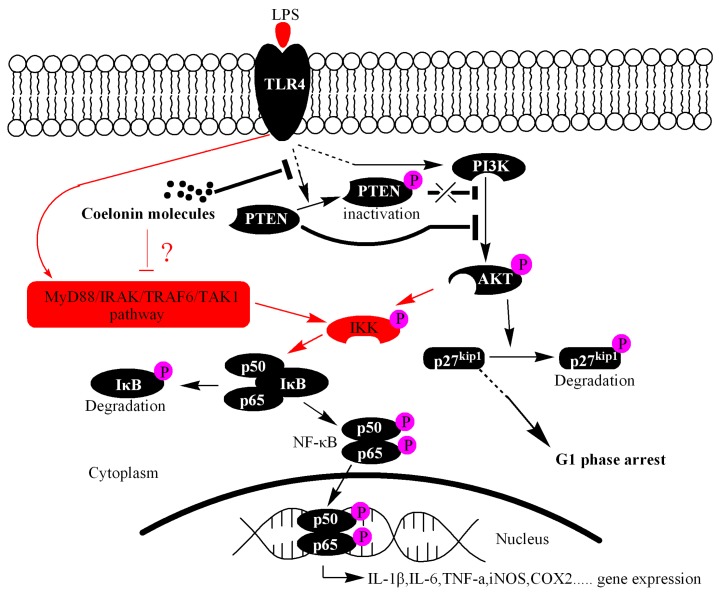
Proposed mechanism of coelonin inhibiting LPS-stimulated activation of NF-κB and inducing G1 phase cell-cycle arrest in RAW264.7 cells. Coelonin inhibits the expression of inflammatory cytokines IL-1β, IL-6 and TNF-α in RAW264.7 cells treated by LPS may partially through the PTEN/AKT pathway. However, it induces G1 cell cycle arrest of RAW264.7 cells by inhibiting the degradation of p27^Kip1^ in a PTEN-dependent manner. The black arrow section has been verified, while the red arrow section needs further validation. The question mark indicated that coelonin may inactivate NF-κB by inhibiting TLR4/MyD88/TRAF6/TAK1 signal pathway, the canonical pathway of NF-κB activation.

In order to better elucidate the potential anti-inflammatory molecular mechanism of coelonin, PEX100 protein microarrays containing 1318 antibodies were used. The results indicated that 32 different phosphorylated proteins were significantly downregulated by pre-treatment with coelonin, which were closely related to the response of LPS-induced signal transduction, immune response and cell proliferation. Additionally, most of these genes were focused on the PI3K/AKT signal pathway, and we performed verification on the phosphorylation levels of PTEN, NF-κB and p27^Kip1^. NF-κB is considered as a central regulator of LPS-induced pro-inflammatory response in macrophage activation [44], which is usually formed as p65:p50 heterodimer. In addition, PEX100 microarray results indicated that the phosphorylation levels of both p65 and p50 were significantly downregulated by coelonin pre-treatment (Table 1), which was further confirmed by western blot and confocal microscopic analysis on p65 nucleus translocation (Figure 6). Studies have shown that numerous dihydrophenanthrene derivatives exert anti-inflammatory effects by inhibiting NF-κB pathway [38,41,42], which was significantly correlated with the presence of phenolic hydroxyl groups [45]. Interestingly, coelonin has two phenolic hydroxyl groups, which is in accordance with its anti-inflammatory effect. However, few reports have studied the upstream targets that regulate the activity of NF-κB by dihydrophenanthrene derivatives. According to the results of the PEX100 microarray, we propose that coelonin may play an anti-inflammatory role by inhibiting the activity of NF-κB through the PTEN/AKT pathway. However, some studies have indicated that the PI3K/AKT pathway negatively regulates LPS induced NF-κB activation [46]. In contrast, other studies have shown that the PI3K/AKT pathway does positively regulate LPS-induced gene expression [27]. We found that LPS stimulation could dramatically induce AKT phosphorylation, IκBa degradation and NF-κB nucleus translocation (Figure 6), and the PI3K inhibitor LY294002 significantly inhibited LPS inducted IL-1β, IL-6 and TNF-α expression in RAW264.7 cells (see Supplementary Figure S5), which is consistent with the findings that the PI3K/AKT pathway is required for LPS induction of gene expression in RAW264.7 cells. Meanwhile, PTEN inhibitor SF1670 significantly increased LPS-induced AKT phosphorylation and cytokines secretion (Figure 7), which is consistent with the report of Zhao et al. [47]. These results indirectly supported that both PI3K/AKT and PTEN/AKT pathways are involved in LPS inducted NF-κB activation. However, the phosphorylation level of Akt did not decrease after co-treatment with PTEN inhibitor SF1670 and coelonin, but coelonin still could significantly down-regulate the expression of inflammatory cytokines induced by LPS. This contradictory result not only indicated that coelonin has other pathways to exert its anti-inflammation activity, but also indicated that AKT pathway has complex mechanisms in the regulation of inflammation. In fact, the exact mechanism of activation of NF-κB by AKT pathway remains controversial. Some reports indicated that activation AKT lead to IKK-dependent IκBα degradation and nucleus translocation of NF-κB, while others shown that AKT-dependent activation of NF-κB by stimulating the transactivation potential of the p65 subunit, rather than inducing IκBα degradation [48]. Obviously, although coelonin could inhibit IκBα phosphorylation and degradation in a dose-dependent manner, it is not clear whether coelonin plays a role through the AKT pathway or TLR4/MyD88/TRAF6/TAK1 pathway, the canonical pathway of NF-κB activation (Figure 9). Therefore, it is difficult to draw a definite conclusion only through the intervention of inhibitors, and more scientific and reasonable experiments need to be designed to elucidate the alternative mechanism of coelonin inactivating NF-κB.

In addition, our study also detected the degradation of p27^Kip1^ after the LPS challenge, which is a downstream target of PTEN/AKT. This effect was dramatically inhibited by coelonin pre-treatment. However, the PTEN inhibitor SF1670 completely abrogated the effect of coelonin (Figure 8). We noticed that LPS treatment also induced RAW264.7 cells G1 phase arrest (Figure 8B), which contradicts a previous report [20], but is consistent with report [49]. The exact molecular mechanism underlying these contradictory results need to be further studied, but it is clear that LPS-induced G1 phase arrest in RAW264.7 cells did not occur through the up-regulation of p27^Kip1^. However, LPS treatment did promote p27^Kip1^ degradation (Figure 8A), and which may be dependent on PTEN inactivation (Figure 9). While coelonin could block the LPS-induced degradation of p27^Kip1^ by restoring PTEN activity (Figure 9). Studies have shown that alveolar macrophage proliferation may play an important role in the formation of multinucleated giant cells and granuloma induced by silica or asbestos [50]. These results suggest that, in addition to inhibiting the secretion of inflammatory factors by macrophages, inhibiting the proliferation of macrophages may also play a role in alleviating silica or asbestos-induced lung pathological changes to an extent.

## 4. Materials and Methods

### 4.1. Active Component Separation, Purification and Identification

A modified polyamide adsorption separation method was used [51]. An amount of 100.0 g tuber powder of *Bletilla striata* (collected from Meichuan, Wuxue, Hubei province, China) was reflux extracted by 1 L 80% ethanol, and the process was repeated three times. The filtrate was concentrated under vacuum to the volume of 600 mL, and an equal volume of distilled water and 30.0 g polyamide (100–200 mesh, Taizhou Luqiao Sijia Biochemical Plastics Factory, Taizhou, China) was added, then the suspension was concentrated under vacuum to the volume of 600 mL. Finally, the mix suspension was packed, and successively eluted with water, 20%, 40%, 60% and 80% ethanol, and each elution was concentrated and dried under vacuum to obtain the fractions F0, F20, F40, F60 and F80, respectively. The IL-1β mRNA expression level, detected by a RT-PCR as the index of anti-inflammation activity on the LPS-induced RAW264.7 cell model was carried out to evaluate the anti-inflammatory effect of the fractions. Then, the active fraction was further separated using silica gel (200–300 mesh, Qingdao Haiyang Chemical Co., Ltd., Qingdao, China) column chromatography, the subfractions were collected after monitoring by TLC (Qingdao Haiyang Chemical Co., Ltd., Qingdao, China), and the anti-inflammation effect was analyzed. The final active compound was purified by Dionex UltiMate™ 3000 semi-prepared HPLC system (Dionex, Sunnyvale, CA, USA) following the guidance of anti-inflammation assay. Furthermore, the molecular weight of the active compounds were determined on an ACQUITY UPLC system coupled to a SYNAPT-G2-Si high-definition mass spectrometer (Waters, Milford, MA USA), and their NMR spectra were measured on a Bruker DRX-600 spectrometer (Bruker, Rheinstetten, Germany) with CD_3_OD as the solvent.

### 4.2. Cell Culture

RAW264.7 cells (ATCC) were cultured in Dulbecco’s Modified Eagle’s Medium (DMEM) containing 10% heat inactivated fetal bovine serum (Gibco, Waltham, MA, USA), 100 units/mL penicillin and 100 μg/mL streptomycin. The cells were grown at 37 °C in a 5% CO_2_ incubator.

### 4.3. RNA Isolation, cDNA Synthesis and RT-PCR

RAW264.7 cells were pre-treated with fractions or active components (dissolved in Dimethyl Sulfoxide, DMSO and diluted with DMEM medium) for 1 h and then stimulated with LPS (200 ng/mL) for 6 h, 0.05% DMSO was applied as the parallel solvent control. Total RNA was extracted using TRIzol (Invitrogen, Carlsbad, CA, USA) according to the manufacturer’s protocol, and subsequently used to obtain cDNA with a reverse transcription polymerase chain reaction following the protocol of PrimeScript reverse transcription reagent kit with genomic DNA (gDNA) Eraser (TaKaRa, Dalian, China) in a 20 μL volume. Levels of IL-1β, IL-6 and TNF-α were determined using specific primers (see Supplementary Table S1) by RT-PCR on a 7500 Real-Time PCR System (Applied Biosystems, Foster City, CA, USA). The expression of each gene was normalized relative to the GAPDH expression level, and relative expression levels were determined using the 2^−∆∆*C*t^ method.

### 4.4. Cytokine Assays

RAW264.7 cells were pre-treated with coelonin for 1 h and then stimulated with LPS (200 ng/mL) for 12 h, 0.05% DMSO was applied as the parallel solvent control. The culture supernatant was collected for IL-6 and TNF-α detection. Then, the remaining cells were treated with 1 mM ATP for an additional 15 min at 37 °C [18], and supernatants were collected for IL-1β detection. IL-6, TNF-α and IL-1β levels were quantified using the cytometric beads array (CBA) method, according to the manufacturer’s protocols (BD, Franklin Lakes, NJ, USA), performed on a BD Accuri™ C6 flow cytometer (BD, Ann Arbor, MI, USA).

### 4.5. Protein Extraction

The cells were washed three times with phosphate-buffered saline (PBS) chilled to 4 °C. Whole-cell proteins were extracted with M-PER Mammalian Protein Extraction Reagent (78503, Thermo Fisher Scientific, Waltham, MA, USA), containing protease and phosphatase inhibitor (Roche, Mannheim, Germany), at 4 °C for 30 min. Then, the samples were centrifuged at 14,000× *g* for 10 min, and the supernatant was transferred to a new tube for analysis. Nuclear proteins were extracted in accordance with the instructions of the Nuclear and Cytoplasmic Protein Extraction Kit (P0027, Beyotime Biotechnology, Shanghai, China) for detection of the activity of NF-κB.

### 4.6. Signal Pathway Phosphorylation Antibody Array Screening

Cell lysates obtained from RAW264.7 cells treated with LPS (200 ng/mL) and with or without coelonin (2.5 μg/mL) were applied to a Phospho Explorer antibody Array PEX100, which was designed and manufactured by Full Moon Biosystems, Inc. (Sunnyvale, CA, USA). The microarray contains 1318 antibodies [52], each of which has two replicates that are printed on a coated glass microscope slide, along with multiple positive and negative controls. The antibody microarray experiment was performed according to the manufacturer’s protocol. The protein phosphorylation level was measured as a ratio of the phospho and unphospho values.

### 4.7. Automated Western Immunoblotting

Before blotting, the protein was quantified using the bicinchoninic acid (BCA) method. Simple western immunoblotting was performed on a simple wes system (ProteinSimple, San Jose, CA, USA) using a Size Separation Master Kit with Split Buffer (12–230 kDa) according to the manufacturer’s standard instruction and using specific antibody. Anti-PTEN (phospho S380) antibody [EP2138Y] (ab76431), Anti-PTEN antibody [Y184] (ab32199), Anti-AKT1 (phospho S473) antibody [EP2109Y] (ab81283), Anti-AKT1/2/3 antibody [EPR16798] (ab179463), Anti-IκBα (phospho S32+S36) antibody (ab12135), Anti-IκBα antibody (ab32518), Anti-NF-κB-p65 antibody (ab32536), Anti-iNOS antibody (ab178945), Anti-COX2 antibody (ab179800) were purchased from Abcam(Cambridge, USA), Anti-p27^kip1^ antibody (2552), Anti-Lamin A/C antibody (2032S,) and anti-β-actin (4970S) antibody were obtained from CST (Danvers, MA, USA). Compass software (version 4.0.0, ProteinSimple, San Jose, CA, USA) was used to program the Peggy Sue and for presentation (and quantification) of the western immunoblots. Output data were displayed from the software calculated average of seven exposures (5–480 s).

### 4.8. Confocal Microscopy Analysis

RAW264.7 cells were grown on glass coverslips. After the treatment with LPS (200 ng/mL) with or without pre-treatment with coelonin or NF-κB-inhibitor APDC, cells were fixed with 4% formalin in PBS for 20 min at room temperature, permeabilized with 0.1% Triton X-100 for 15 min, and nonspecific protein binding sites were blocked with 10% FBS at room temperature for 1 h. Then samples were incubated overnight at 4 °C with a p65 (ab32536, Abcam, Cambridge, MA, USA) specific Antibody (1:100 dilution in PBS), washed three times with PBS, and then incubated with a Alexa Fluor^®^ 488-labeled secondary antibody (ab60314, Abcam; 1:600 dilution in PBS) for 2 h at room temperature. The coverslips were rinsed three times with PBS, then stained with 4′,6-diamidino-2-phenylindole (DAPI) for 10 min. Again, the coverslips were rinsed with PBS three times and then mounted on glass slides using Antifluorescence Quenching Sealing Solution. The coverslips were analyzed by confocal microscopy (LSM880; ZEISS, Upper Cohen, Germany).

### 4.9. Cell Cycle Analysis

After treatment, the RAW264.7 cells were washed three times with chilled 1 × PBS (pH 7.4) and fixed overnight with 70% ethanol at 4 °C followed by centrifugation at 2,000 rpm for 8 min. The cells were re-suspended in 1 × PBS (pH 7.4) with PI/RNase Staining Buffer (BD, 550825, San Diego, CA, USA) for 30 min. The cell cycle distributions were analyzed on a BD Accuri™ C6 flow cytometer (BD, Ann Arbor, MI, USA).

### 4.10. Statistical Analysis

Data are presented as the mean ± SD derived from at least three independent experiments. ANOVA analysis was used to examine the statistical significance of the differences between the groups, and the criterion for significance for all the experiments was *p* < 0.05.

## 5. Conclusions

Our study indicated that coelonin is one of the active components of *Bletilla striata*. Furthermore, we showed that using a PEX100 antibody microarray, a total of 32 different phosphorylation proteins were downregulated by coelonin pre-treatment on LPS-induced RAW264.7 cells. The maximum number of proteins belonged to the PI3K/AKT signal pathway, and three of them, PTEN, p65 and p27 ^Kip1^ were confirmed by western blot, and more proteins and signaling pathways need to be verified. Western blot and confocal microscopy analysis revealed that coelonin inhibits the expression of IL-1β, IL-6 and TNF-α in a dose-dependent manner by eliminating lipopolysaccharide-induced NF-κB activity. However, besides inhibiting IκBα degradation, which pathways coelonin mainly inhibits the activation of NF-κB still need to be further studied. While, we did confirm that coelonin inhibit LPS-induced p27 ^Kip1^ degradation and block RAW264.7 cells in the G1 phase in a PTEN dependent manner (Figure 9). Overall, our results suggest that traditional Chinese medicine *Bletilla striata* has anti-inflammatory activity, and coelonin is one of the main active components. It may play a potential role in treating silicosis by inhibiting the proliferation of macrophages and the secretion of inflammatory factors. Additionally, PTEN may play an important role during this process, our following work will use modified RAW264.7 cells to address this issue.

## Figures and Tables

**Table 1:** The phosphorylation of 32 proteins were significantly downregulated by coelonin treatment (*p* < 0.05).

Name	Phosphorylation Site or Antibody	Gene ID	Name	Phosphorylation Site or Antibody	Gene ID
HSP90B	Ser254	3326	Trk A	Tyr791	4914
NFκB-p65	Ser536	5970	PKC α/β II	Ab-638	5578
Ezrin	Thr566	7430	MAP3K8/COT	Thr290	1326
FLT3	Ab-599	2322	NFκB-p105/p50	Ab-932	4790
p53	Ser33	7157	p27^Kip1^	Thr187	1027
CK2-b	Ab-209	1460	Shc	Tyr349	6464
ATF1	Ab-63	466	Smad1	Ab-187	4086
AXL	Tyr691	558	ERK3	Ab-189	5597
Rac1/cdc42	Ser71	5879	Caveolin-1	Tyr14	857
PTEN	Ser380	5728	MARCKS	Ser163	4082
CDK2	Ab-160	1017	GRK2	Ser685	156
DNA-PK	Ab-2056	5591	Ephrin B1	Ab-317	1947
EGFR	Ab-1069	1956	Estrogen Receptor-α	Ser104	2099
Keratin 8	Ser73	3875	BRCA1	Ser1524	672
ATPase	Ab-16	476	PTEN	Ser380/Thr382/Thr383	5728
CDC25A	Ab-75	993	eIF4B	Ser422	1975

## Supplementary Materials

Supplementary materials can be found at https://www.mdpi.com/1422-0067/20/18/4422/s1.

## Abbreviations

TLR4	Toll-like receptor 4
MyD88	myeloid differentiation factor 88
IRAK	IL-1 receptor associated kinase
TRAF6	TNF receptor associated factor 6
TAK1	TGF beta-Activated Kinase 1
IKKs	inhibitor of nuclear factor-κB kinase
PTEN	phosphatase and tensin homologue on chromosome ten
PI3K	phosphatidylinositol-3-kinases
AKT	v-akt murine thymoma viral oncogene homolog
p27Kip1	cyclin dependent kinase inhibitor 1B
NF-κB	nuclear factor-kappa B
IκBa	inhibitor of NF-κB
IL-1β	interleukin-1β
iNOS	inducible nitric oxide synthase
COX2	cyclooxygenase 2

## References

[1] Pérez G., Martha R. (2010). Orchids: A review of uses in traditional medicine, its phytochemistry and pharmacology. J. Med. Plants Res..

[2] Sun D.F., Shi J.S., Zhang W.M., Gu G.P., Zhu C.L. (2006). Study on the extraction of polysaccharides from *Blettila striata* by the continuous counter-current equipment (in Chinese). Chin. Wild Plant. Resour..

[3] Lv X.B., Huang C.Q., Wu Z.C., Yang D.J., Den L. (2012). The therapeutic effects of polysaccharides from *Bletilla striata* on gastric ulcer rats. J. Yunnan Univ. Tradit. Chin. Med..

[4] Luo Y., Diao H.J., Xia S.H., Dong L., Chen J.N., Zhang J.F. (2010). Physiologically active polysaccharide hydrogel promotes wound healing. J. Biomed. Master Res. A.

[5] Dong Y.X., Liu X.X., Dong L., Wang X., Huang Y., Wang Y.L. (2016). Study on hemostatic effect and mechanism of polysaccharides from *Bletilla striata* in blood heat and hemorrhage model rats. China Pharm..

[6] Zhang Y., Zhou Q., Lai S. (2009). The effects of *Bletilla striata* polysaccharide on proliferation of hematopoietic cells and immunological function in mice treated by cyclophosphamide. Pharmacol. Clin. Chin. Mater. Med..

[7] Liu X.X. (2015). Hemostatic effects, mechanism and biopotency of *Bletillae rhizoma*. Master’s Thesis.

[8] Shi Z.Z., Xu Z.H., Fu Y.H., Yu H.S., Jiang F.S., Ding Z.S. (2015). Study of *Rhizoma bletillae* fibrous root alcohol extract on anti gastric ulcer. J. Shanxi Coll. Tradit. Chin. Med..

[9] Deng Y.Z., Jin L.X., Gao C.X., Qian C.D., Jiang F.S., Ding Z.S., Li M.Y. (2016). Research on the Anti-Pulmonary Fibrosis effect of the small molecule components of *Bletilla striata* in rat silicosis model. J. Chin. Med. Mater..

[10] Li H.Y., Shi Z.Z., Shu L.F., Wang J., Li M.Y., Ding Z.S., Jiang F.S. (2016). Research on the Anti-Pulmonary Fibrosis effect of the *Bletilla striata* polysaccharide in rat silicosis model. J. Chin. Med. Mater..

[11] Young K.S., Woo P.S., Ran L.M., Young K.E., Taek U.S., Hoon K.Y., Sik P.C., Bal L.H. (1998). Silica induced expression of IL-1β, IL-6, TNF-β, TGF-α, in the experimental murine lung fibrosis. Tuberc. Respir. Dis..

[12] Piguet P.F., Vesin C., Grau G.E., Thompson R.C. (1993). Interleukin 1 receptor antagonist (IL-1ra) prevents or cures pulmonary fibrosis elicited in mice by bleomycin or silica. Cytokine.

[13] Piguet P.F., Collart M.A., Grau G.E., Sappino A.P., Vassalli P. (1990). Requirement of tumour necrosis factor for development of silica-induced pulmonary fibrosis. Nature.

[14] Smoktunowicz N., Alexander R.E., Franklin L., Williams A., Holman B., Mercer P. (2015). The anti-fibrotic effect of inhibition of TGFβ-ALK5 signalling in experimental pulmonary fibrosis in mice is attenuated in the presence of concurrent γ-herpesvirus infection. Dis. Model. Mech..

[15] Yang J.Z., Jiang H., Wang W.J., Zhang Y.M., Liu Y., Chen Y.G. (2014). Isolation and Characterization of Batatasin III and 3,4′-Dihydroxy-5-methoxybibenzyl: A Pair of Positional Isomers from *Sunipia scariosa*. Trop. J. Pharm. Res..

[16] Zhang H.L., Tian L., Fu H.W., Pei Y.H., Hua H.M. (2005). Studies on constituents from the fermentation of Alternalia sp.. China J. Chin. Mater. Med..

[17] Deng Y.Z., Jin L.X., Gao C.X., Qian C.D., Jiang F.S., Ding Z.S., Li M.Y. (2016). Study on the Active Components and Molecular Mechanism of *Bletilla striata* on Suppressing Pulmonary Fibrosis. J. Chin. Med. Mater..

[18] Stoffels M., Zaal R., Kok N., Van der Meer J.W., Dinarello C.A., Simon A. (2015). ATP-Induced IL-1β Specific Secretion: True Under Stringent Conditions. Front. Immunol..

[19] Beutler B., Jiang Z., Georgel P., Crozat K., Croker B., Rutschmann S., Du X., Hoebe K. (2006). Genetic analysis of host resistance: Toll-like receptor signaling and immunity at large. Annu. Rev. Immunol..

[20] Jiao H.W., Jia X.X., Zhao T.J., Rong H., Zhang J.N., Cheng Y., Zhu H.P., Xu K.L., Guo S.Y., Shi Q.Y. (2016). Up-regulation of TDAG51 is a dependent factor of LPS-induced RAW264.7 macrophages proliferation and cell cycle progression. Immunopharm. Immunot..

[21] Zhu Y., Tong Q., Ye J., Ning Y., Xiong Y., Yang M., Xiao H., Lu J., Xu W., Li J. (2017). Nogo-B Facilitates LPS-Mediated Immune Responses by Up-Regulation of TLR4-Signaling in Macrophage RAW264.7. Cell Physiol. Biochem..

[22] Surh Y.J., Chun K.S., Cha H.H., Han S.S., Keum Y.S., Park K.K., Lee S.S. (2001). Molecular mechanisms underlying chemopreventive activities of anti-inflammatory phytochemicals: Down-regulation of COX-2 and iNOS through suppression of NF-kappa B activation. Mutat. Res..

[23] Wang Q.S., Xiang Y., Cui Y.L., Lin K.M., Zhang X.F. (2012). Dietary Blue Pigments Derived from Genipin, Attenuate Inflammation by Inhibiting LPS-Induced iNOS and COX-2 Expression via the NF-κB Inactivation. PLoS ONE.

[24] Cohen G., Tretiakova M., Carroll R., Bissonnette M. (2003). COX-2 and iNOS are overexpressed in human colonic aberrant crypt foci. Gastroenterol.

[25] Pan M.H., Lai C.S., Wang Y.J., Ho C.T. (2006). Acacetin suppressed LPS-induced up-expression of iNOS and COX-2 in murine macrophages and TPA-induced tumor promotion in mice. Biochem. Pharmacol..

[26] Lu Y.C., Yeh W.C., Ohashi P.S. (2008). LPS/TLR4 signal transduction pathway. Cytokine.

[27] Ojaniemi M., Glumoff V., Harju K., Liljeroos M., Vuori K., Hallman M. (2003). Phosphatidylinositol 3-kinase is involved in Toll-like receptor 4-mediated cytokine expression in mouse macrophages. Eur. J. Immunol..

[28] He Z.Y., Gao Y., Deng Y.X., Li W., Chen Y.M., Xing S.P., Zhao X.Y., Ding J., Wang X.R. (2012). Lipopolysaccharide Induces Lung Fibroblast Proliferation through Toll-Like Receptor 4 Signaling and the Phosphoinositide3-Kinase-Akt Pathway. PLoS ONE.

[29] Zhang L., Huang C.Q., Guo Y.J., Gou X.X., Hinsdale M., Lloyd P., Liu L. (2015). MicroRNA-26b Modulates the NF-kB Pathway in Alveolar Macrophages by Regulating PTEN. J. Immunol..

[30] Yang Z., Cao X.M., Xu W.T., Xie C., Chen J., Zhu Y., Lu N.H. (2018). Phosphorylation of phosphatase and tensin homolog induced by Helicobacter pylori promotes cell invasion by activation of focal adhesion kinase. Oncol. Lett..

[31] Feng X.J., Liu S.X., Wu C., Kang P.P., Liu Q.J., Hao J., Li F., Zhang Y.J., Fu X.H., Zhang S.B. (2014). The PTEN/PI3K/Akt signalling pathway mediates HMGB1-induced cell proliferation by regulating the NF-κB/cyclin D1 pathway in mouse mesangial cells. Am. J. Physiol. Cell Physiol..

[32] Cantley L.C. (2002). The phosphoinositide 3-kinase pathway. Science.

[33] Prasad S.B., Yadav S.S., Das M., Modi A., Kumari S., Pandey L.K., Singh S., Pradhan S., Narayan G. (2015). PI3K/AKT pathway-mediated regulation of p27Kip1is associated with cell cycle arrest and apoptosis in cervical cancer. Cell Oncol..

[34] Liang J., Slingerland J.M. (2003). Multiple roles of the PI3K/PKB (Akt) pathway in cell cycle progression. Cell Cycle.

[35] Li J., Yang X.K., Yu X.X., Ge M.L., Wang W.L., Zhang J., Hou Y.D. (2000). Overexpression of p27(KIP1) induced cell cycle arrest in G1 phase and subsequent apoptosis in HCC-9204 cell line. World J. Gastroenterol..

[36] Kuo M.Y.P., Hsu H.Y., Kok S.H., Kuo R.C., Yang H., Hahn L.J., Chiang C.P. (2002). Prognostic role of p27Kip1expression in oral squamous cell carcinoma in Taiwan. Oral Oncol..

[37] Mamillapalli R., Gavrilova N., Mihaylova V.T., Tsvetkov L.M., Wu H., Zhang H., Sun H. (2001). PTEN regulates the ubiquitin-dependent degradation of the CDK inhibitor p27^KIP1^ through the ubiquitin E3 ligase SCF^SKP2^. Curr. Biol..

[38] Kovács A., Vasas A., Hohmann J. (2008). Natural phenanthrenes and their biological activity. Phytochemistry.

[39] He X., Fang J.C., Wang X.X., Zhao Z.F., Chang Y., Guo H., Zheng X. (2017). *Bletilla striata*: Medicinal uses, phytochemistry and pharmacological activities. J. Ethnopharmacol..

[40] Qian C.D., Jiang F.S., Yu H.S., Shen Y., Fu Y.H., Cheng D.Q., Gan L.S., Ding Z.S. (2015). Antibacterial Biphenanthrenes from the Fibrous Roots of *Bletilla striata*. J. Nat. Prod..

[41] Lin Y., Wang F., Yang L., Chun Z., Bao J., Zhang G. (2013). Anti-inflammatory phenanthrene derivatives from stems of Dendrobium denneanum. Phytochemistry.

[42] Ma W., Zhang Y., Ding Y.Y., Liu F., Li N. (2015). Cytotoxic and anti-inflammatory activities of phenanthrenes from the medullae of *Juncus effusus* L.. Arch. Pharm. Res..

[43] Jiang F.S., Shen X.T., Ding B., Li M.Y., Ding Z.S., Lv G.Y. (2019). Comparison of the contents of three active ingredients in *Bletilla striata* from different sources. China J. Chin. Mater. Med..

[44] Lawrence T., Natoli G. (2011). Transcriptional regulation of macrophage polarization: Enabling diversity with identity. Nat. Rev. Immunol..

[45] Kanekar Y., Basha K., Duche S., Gupte R., Kapat A. (2013). Regioselective synthesis of phenanthrenes and evaluation of their anti-oxidant based anti-inflammatory potential. Eur. J. Med. Chem..

[46] Luyendyk J.P., Schabbauer G.A., Tencati M., Holscher T., Pawlinski R., Mackman N. (2008). Genetic Analysis of the Role of the PI3K-Akt Pathway in Lipopolysaccharide-Induced Cytokine and Tissue Factor Gene Expression in Monocytes/Macrophages. J. Immunol..

[47] Zhao M., Zhou A., Xu L., Zhang X. (2014). The role of TLR4-mediated PTEN/PI3K/AKT/NF-κB signaling pathway in neuroinflammation in hippocampal neurons. Neuroscience.

[48] Mayo M.W., Madrid L.V., Westerheide S.D., Jones D.R., Yuan X.J., Baldwin A.S., Whang Y.E. (2002). PTEN Blocks Tumor Necrosis Factor-induced NF-κB-dependent Transcription by Inhibiting the Transactivation Potential of the p65 Subunit. J. Biol. Chem..

[49] Vadiveloo P., Keramidaris E., Morrison W., Stewart A. (2001). Lipopolysaccharide-induced cell cycle arrest in macrophages occurs independently of nitric oxide synthase II induction. BBA - Mol. Cell Res..

[50] Prieditis H., Adamson I.Y.R. (1996). Alveolar macrophage kinetics and multinucleated giant cell formation after lung injury. J. Leukocyte Biol..

[51] Xu M., Shen Y., Zhang K., Liu N.N., Jiang F.S., Ding Z.S. (2013). Antioxidant activity of total flavonoid aglycones and the main compound pinostrobin chalcone separated from leaves of Carya cathayensis. Chin. J. ETMF.

[52] Mitchell S.J., Martin-Montalvo A., Mercken E.M., Palacios H.H., Ward T.M., Abulwerdi G., Minor R.K., Vlasuk G.P., Ellis J.L., Sinclair D.A. (2014). The SIRT1 activator SRT1720 extends lifespan and improves health of mice fed a standard diet. Cell Rep..

