# Supplementary material for: Coelonin, an Anti-Inflammation Active Component of Bletilla striata and Its Potential Mechanism
Source: Int J Mol Sci. 2019 Sep 8;20(18):4422. doi: 10.3390/ijms20184422 (PMC6770560; doi:10.3390/ijms20184422)
Supplement: Supplementary file 1 [file ijms-20-04422-s001.pdf]

Supplementary Table S1 Primers for specific genes

Oligo Name	Sequence（5′to3′）	Length（bp）
IL-1β-F1	5′-caa atc tcg cag cag cac atc-3′	21
IL-1β-R1	5′-tca tct cgg agc ctg tag tgc-3′	21
IL-6-F1	5′-agt tgc ctt ctt ggg act ga-3′	20
IL-6-R1	5′-ttc tgc aag tgc atc atc gt-3′	20
TNF-α-F1	5′-cga gtg aca agc ctg tag ccc-3′	21
TNF-α-R1	5′-ggg cag cct tgt ccc ttg a-3′	19
GAPDH-F1	5′-cat cac tgc cac cca gaa gac t-3′	22
GAPDH-R1	5′-gac aca ttg ggg gta gga aca c-3′	22

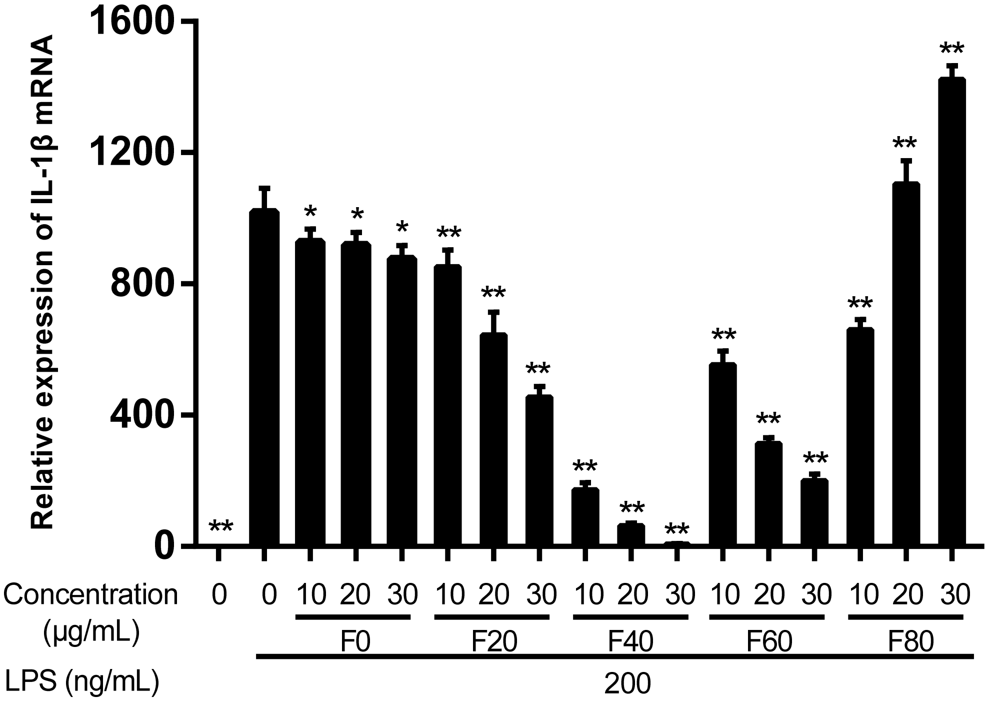

Supplementary Figure S1. Relative expression of IL-1β mRNA after treatment with F40. RAW264.7 cells were pretreated with different concentration of F0-80 for 1 hour and then treated with 200 ng/mL LPS for 6 h, 0.05% DMSO was applied as the parallel solvent control. Total RNA was extracted and genes expression level were analysed by RT-PCR in triplicate. The expression level of each gene was normalized to GAPDH mRNA. Data are expressed as mean ± SD (n=6). **P* < 0.05, ** *P* < 0.01 vs. LPS treatment group.

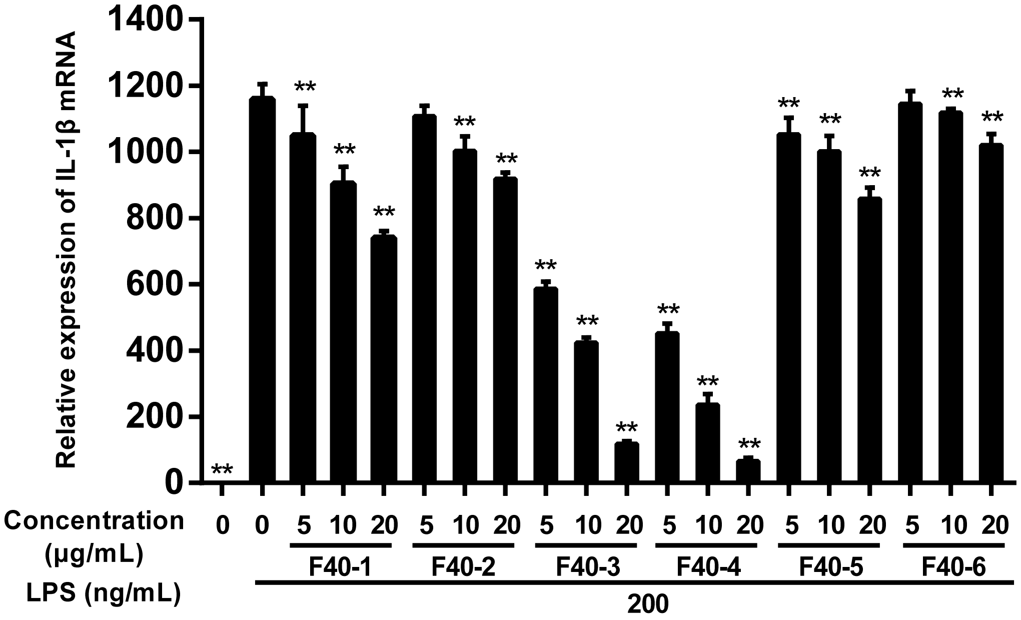

Supplementary Figure S2. The relative expression of IL-1β mRNA after treatment with sub-fractions of F40-1- F40-6. RAW264.7 cells were pretreated with different concentration of different sub-fractions of F40 for 1 h, then treated with 200 ng/mL LPS for 6 h, 0.05% DMSO was applied as the parallel solvent control. Total RNA was extracted and genes expression levels were analysed by RT-PCR in triplicate. The expression level of each gene was normalized to GAPDH mRNA. Data are expressed as mean ± SEM. ** $P < 0.01$ vs LPS treatment group.

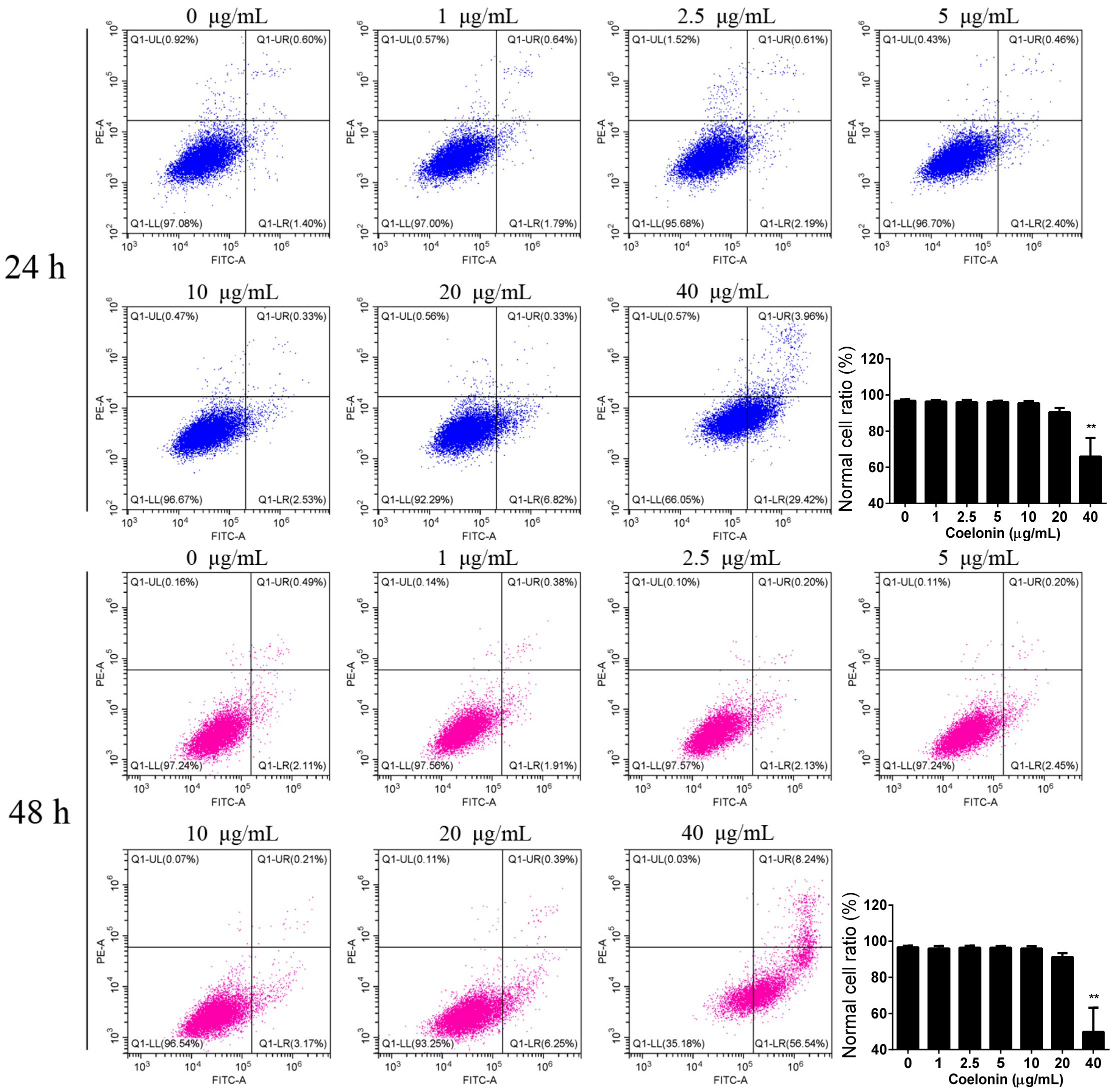

Supplementary Figure S3. Coelonin treatment did not cause any obvious cell death. RAW264.7 cells were treated with increasing doses of coelonin for 24 h and 48 h, 0.05% DMSO was applied as the parallel solvent control. Apoptotic cells were detected using Annexin V-FITC apoptosis detection kit (BD Pharmingen 556547，SanDiego, CA, USA) and analyzed by flowcytometry on a Cytoflex S flow cytometer (Beckman Coulter, USA). Data are expressed as mean ± SD (n = 3). ** P<0.01 vs solvent control group. No significant cell death was observed in all treatment groups. Except for 40 μg/mL treatment group, significant apoptosis was observed at 24 h and 48 h, there was no significant difference in apoptosis among other treatment groups.

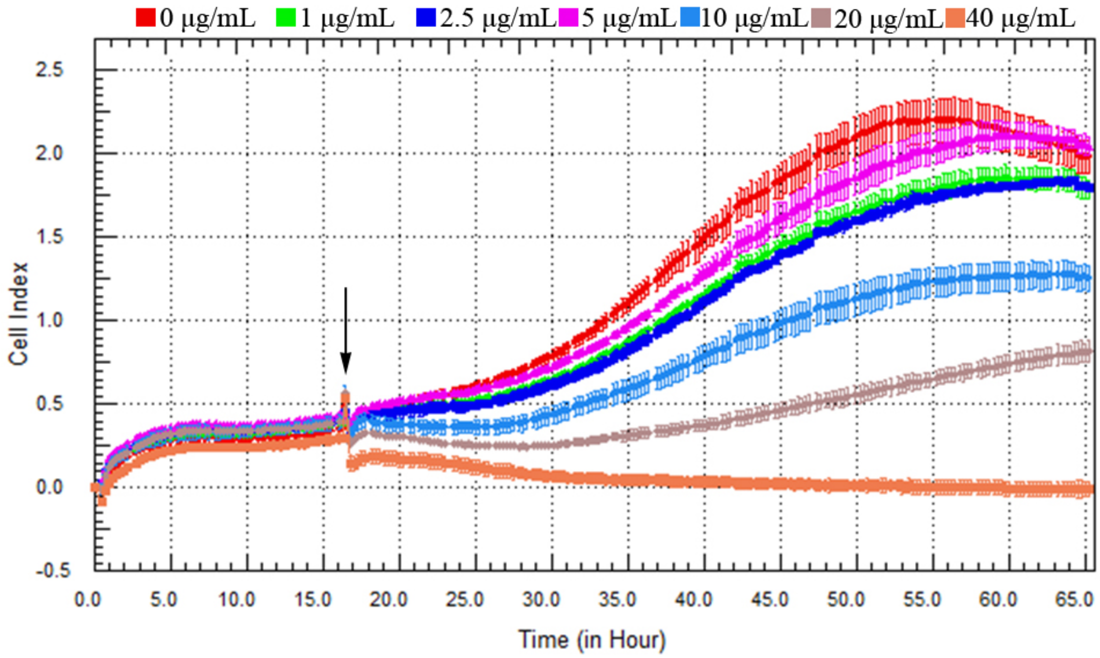

Supplementary Figure S4. The effect of coelonin on the growth of RAW264.7 cells. 150 μL of suspension of 50 000 RAW264.7 cells were seeded into 16-well of the E-plates, the cell index was recorded every 15 min for about 17 h, then cells were exposed to (black arrow) increasing doses of coelonin and the cell index read for additional 48 h on a X celligence system (ACEA biosciences, Inc., California, San Diego, USA), 0.05% DMSO was applied as the parallel solvent control. Data are expressed as mean ± SD (n = 4). Apoptotic analysis showed that Coelonin had no obvious cytotoxicity in the dose range of 40 μg/mL. Therefore, cell index results indicated that coelonin mainly inhibited cell proliferation. The concentration of 10 and 20 μg/mL showed significant proliferation inhibition, while 40 μg/mL completely inhibited cell proliferation and significantly induced cell apoptosis. Relatively, the concentration below 5 μg/mL had no significant inhibitory activity.

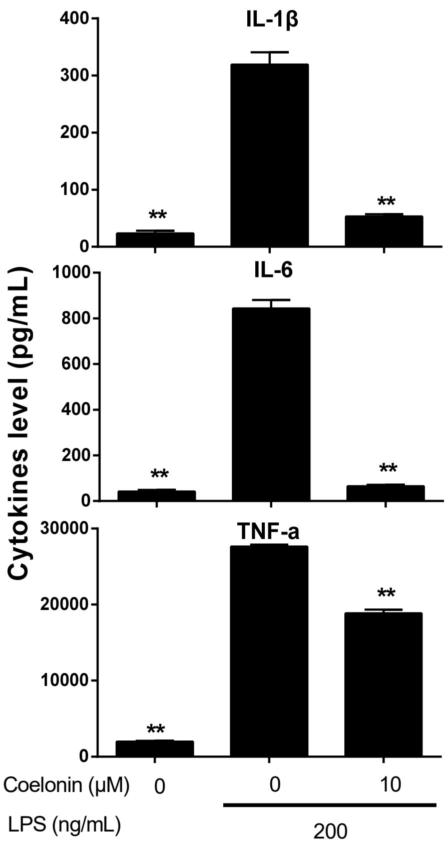

Supplementary Figure S5. PI3K inhibitor LY294002 significantly inhibited LPS inducted IL-1β, IL-6 and TNF-*α* expression in RAW264.7 cells. RAW264.7 cells were pretreated with LY294002 (10 μM) for 1 hour and then treated with 200 ng/mL LPS, 0.05% DMSO was applied as the parallel solvent control. 6 h after LPS stimulation, culture supernatants were collected for IL-6 and TNF-*α* detection by CBA method; for IL-1β detection the remaining cells were following treated by 1 mM ATP for additional 15 min at 37 ℃, then supernatants were collected and analysed by CBA method. Data are expressed as mean ± SD (n=6). ** *P*<0.01 vs. LPS treatment group.
